# Photoassisted Chemical Transformation of Cu_2_O Nanooctahedra into Cu_2_S Quantum-Dot Superstructures: Structural and Photoelectrochemical Properties

**DOI:** 10.1021/acsmaterialsau.5c00106

**Published:** 2025-08-13

**Authors:** Dávid Kovács, György Z. Radnóczi, Zsolt E. Horváth, Krisztina Frey, Attila Sulyok, Zsolt Fogarassy, József S. Pap, András Deák, Dániel Zámbó

**Affiliations:** † Institute of Technical Physics and Materials Science, 303347 HUN-REN Centre for Energy Research, Konkoly-Thege M. út 29-33., H-1121 Budapest, Hungary; ‡ Institute of Energy Security and Environmental Safety, HUN-REN Centre for Energy Research, Konkoly-Thege M. út 29-33., H-1121 Budapest, Hungary; § Department of Physical Chemistry and Materials Science, Budapest University of Technology and Economics, Műegyetem rkp. 3., H-1111 Budapest, Hungary

**Keywords:** copper sulfide, quantum dot, superstructure, sulfidation, photoelectrochemistry

## Abstract

Copper sulfides represent a broad range of chemical compounds, including naturally occurring minerals and wet-chemically synthesized nanoparticles. Tailoring the size, shape, and chemical composition of Cu_2‑*x*_S nanoparticles enables the tuning of their optical and electronic properties allowing the switch between semiconducting and plasmonic characteristics. While the sulfidation of metals and metal oxides can even occur spontaneously under ambient storage conditions, the targeted synthesis of Cu_2‑*x*_S nanoparticles mostly relies on the use of inorganic sulfur compounds. Inspired by the natural sulfidation reactions, a novel approach is developed in this paper to transform sacrificial Cu_2_O nanooctahedra by a short-chain organic thiol (β-mercaptoethanol) into spherical Cu_2_S superstructures consisting of phase-pure Cu_2_S quantum dots. The optical and photoelectrochemical properties are thoroughly investigated and supplemented by advanced electron microscopy analysis to identify the phase of the superstructure building blocks. Structural and surface analyses reveal that the superstructures are composed of small (4–5 nm) Cu_2_S quantum dots spatially separated by a thin amorphous ligand layer. The results highlight the dual role of β-mercaptoethanol serving both as a sulfur source and as a stabilizing ligand upon superstructure formation. To synthesize semiconductor/metal multicomponent nanostructures, the surface of the superstructures is decorated with Au nanograins initiated by the photoreduction of aqueous Au^3+^ ions. Upon the fabrication of working electrodes from the developed superstructures, the p-type nature of the Cu_2_S is demonstrated by open-circuit potentiometry. Superstructures supply negative photocurrent under UV irradiation, which can be further enhanced by the presence of Au nanograins. Using the developed synthetic method, phase-pure photofunctional nanomaterials can be prepared by the sulfidation of cuprous oxide in a controlled manner.

## Graphical abstract

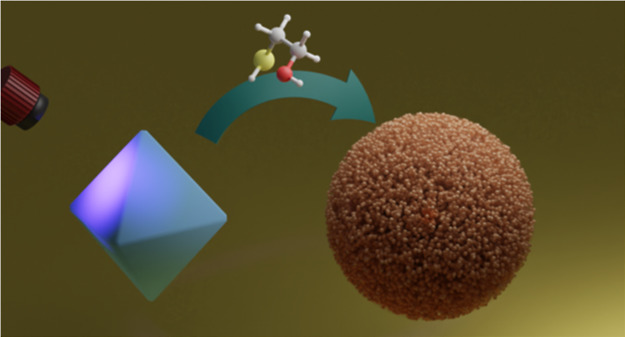

## Introduction

Among p-type copper-based semiconductors, cuprous oxide (Cu_2_O) and copper sulfide (Cu_2‑*x*_S) have attracted significant interest due to their unique optical, structural, and catalytic properties. In addition to the precise control over the morphology and the corresponding optical features of Cu_2_O nanoparticles (NPs), Cu_2‑*x*_S nanocrystals deliver additional benefits, as their stoichiometry can be tuned to switch between plasmonic (x→1) and semiconducting (x→0) properties.^1,2^

Colloidal Cu_2_O nanocrystals can be synthesized in a wide range of well-defined shapes, including spheres, cubes, octahedra, rods, and hollow polyhedra, by a suitable choice of precursors and synthesis conditions.^3,4^ Among these, the octahedral morphology shows novel facet-dependent optical and electronic properties and enhanced photocatalytic performance associated with the ⟨111⟩ facets of the particles.^5^ Similarly, recently developed wet chemical synthesis methods of Cu_2‑*x*_S nanoparticles enabled a precise tuning of the morphology and composition that facilitated their application as a solar energy harvester,^6–8^ water splitting cocatalyst,^9^ supercapacitor,^10^ and catalyst for CO_2_ reduction.^11^ While these methods mostly rely on the reduction of the copper salt in the presence of a sulfur source and stabilizers,^12^ the transformation of Cu, Cu_2_O, or CuO into Cu_2‑*x*_S can also be initiated by adding inorganic sulfidation agents, such as H_2_S, Na_2_S, or NaSH, exploiting the Kirkendall effect to obtain hollow nanoparticles with application potential in hydrogen generation or even sensing.^13–16^ Also, photofunctional properties can be further extended by combining Cu_2‑*x*_S particles with noble metals (e.g., Au) being reduced from its aqueous salt by the photogenerated carriers in the semiconductor.^17^

Sulfidation in general, refers to a chemical transformation process of metal-based materials upon their interaction with a sulfur-containing species in various oxidation states. This process is particularly relevant for materials containing chalcophile metals (such as copper), which readily interact with sulfur to form metal sulfides.^18^ Copper sulfide exists in multiple distinct crystalline phases ranging from stoichiometric Cu_2_S (chalcocite) to CuS (covellite), and other, nonstoichiometric varieties (Cu_2‑*x*_S).^19^ While the use of well-defined Cu_2_O or CuO nanoparticles as a sacrificial template and a suitable sulfur-containing compound (inorganic sulfides) led to the formation of promising stoichiometries (such as Cu_7_S_4_) with advanced photofunctional properties,^20,21^ producing phase-pure Cu_2_S nanoparticles with prolonged stability is challenging,^22^ due to the coexistence of metastable phases.

Although the chemical concept of sulfidation of a sacrificial template is based on the introduction of a sulfur source to the NPs during the synthesis, it has to be emphasized that this process can also occur naturally, due to the presence of vast varieties of sulfur-containing compounds in the atmosphere^23^ or even in a cellular environment.^24^ Anthropogenic emission and microbial reactions have increased the concentration of organic and inorganic sulfur species to an extent which can initiate ambient corrosion of metals and metal oxides.^25–27^ While the role of organic surface- and crystal-bound thiols have already been thoroughly investigated in Cu_2‑*x*_S model systems,^28^ the direct wet-chemical sulfidation of sacrificial cuprous oxide templates with thiols is much less discussed in the literature. In contrast, studies rather focus on the surface of the particles and show that the interaction with alkanethiols and oxidized copper surfaces leads to the reduction of CuO/Cu_2_O to metallic copper,^29^ while the thiol is oxidized to disulfide.^30^ Zhang et al. reported on the partial sulfidation of Cu_2_O/CuS core/shell nanocubes by sonicating Cu_2_O in the presence of thioacetamide demonstrating that short-chain thiolated compounds can facilitate an in situ anion exchange reaction.^15^ Note that thioacetamide has been utilized in the microwave- and conventional hydrothermal synthesis of morphologically diverse CuS particles because the release of H_2_S via solution chemistry can be controlled.^31,32^ However, due to the large variety of organic sulfur compounds existing in the atmospheric environment, understanding of the interaction between Cu_2_O and thiols is of great importance to design durable and stable photocatalysts.

Previously, we have developed a robust wet-chemical method to synthesize octahedral Cu_2_O NPs via the rapid reduction of aqueous Cu­(OH)_2_ precipitates using hydrazine.^33^ Under appropriate storage conditions (ethanolic medium, 4 °C, dark), these particles exhibit excellent long-term stability. Nevertheless, as will be shown later, after prolonged storage periods (beyond 8 months), signs of surface degradation were observed which could not be linked to the classical overoxidation of the Cu_2_O but rather point toward the partial sulfidation of the surface. To understand the underlying process and to mimic the same effect experimentally, changes in the morphological, compositional, optical, and photoelectrochemical properties of Cu_2_O nanooctahedra are investigated in this study using a short-chain thiol as a model sulfur source. We demonstrate that the controlled sulfidation of well-defined Cu_2_O nanooctahedra can be initiated by introducing a low amount of β-mercaptoethanol (BME), which is the closest sulfur-containing structural analogue of the stabilizing molecules (ethanol) of the colloidal particles. The effect of the sulfidation agent’s concentration and the conditions (time, solvent, and light exposure) on the evolution of the copper sulfide phase are thoroughly investigated. Upon optimizing the sulfidation process, the precursor Cu_2_O octahedra can be transformed into Cu_2_S quantum-dot superstructures, whose structural, optical, and photoelectrochemical properties are shown. This platform is also used to prepare Au@Cu_2_S heterostructures by the photoassisted reduction of the gold salt in an aqueous medium.

## Experimental Section

### Chemicals

Hydrogen tetrachloroaurate trihydrate (HAuCl_4_·3H_2_O, trace metals basis), copper­(II) nitrate trihydrate (Cu­(NO_3_)_2_·3H_2_O), sodium hydroxide (NaOH), β-mercaptoethanol (BME) and buffer solution (boric acid/potassium chloride/sodium hydroxide, pH 9) were purchased from Merck (Sigma Aldrich). Hydrazine hydrate (N_2_H_4_·H_2_O) was supplied by Thermo Scientific. Methanol, ethanol, and 2-propanol were purchased from VWR International. Ultrapure water (0.055 μS cm^−1^) was prepared using a Sartorius Arium Mini Plus device. All glassware and stirring bars were cleaned with aqua regia (3: 1 V/V ratio of cc. HCl and HNO_3_), rinsed thoroughly with ultrapure water, and dried at 70 °C for 6 h. Quartz cuvettes were stored in 2 V/V % Hellmanex II solutions, rinsed with ultrapure water and 2-propanol followed by drying under N_2_ flow.

### Synthesis of Cu_2_O Nanoparticles

The wet-chemical synthesis of Cu_2_O nanooctahedra followed a slightly modified version of a protocol published in our previous study.^33^ In a 100 mL glass vial, 1 mL of 0.1 M Cu­(NO_3_)_2_ solution was added to 91.8 mL of ultrapure water under mild stirring. Subsequently, 200 μL of a 1 M NaOH solution was injected, turning the solution blue upon the formation of Cu­(OH)_2_. After 30 s, 3.5 mL of a freshly prepared 0.2 M N_2_H_4_ solution was added dropwise using a transfer pipette (ca. 1 droplet/s) under vigorous stirring. After approximately 25 droplets, the solution gradually turned greenish-yellow and then orange, indicating the formation of Cu_2_O nanoparticles. The mixture was stirred for an additional 10 min and then centrifuged at 6500 rcf for 10 min. The precipitate was redispersed in a 1:1 mixture of EtOH and ultrapure water. This step was repeated once again to remove all of the unreacted components. Finally, after another centrifugation, the product was redispersed in 10 mL of abs. EtOH and stored in a refrigerator (4 °C, dark–referred to as an ethanolic stock solution in the following, Figure S1). The concentration of Cu_2_O in the stock solution is ca. 0.5 g L^–1^.

## Synthesis of Cu_2_S Quantum Dot Superstructures

In a 44 mL sealed glass vial, 1 mL of ethanolic Cu_2_O stock solution was added to a mixture of 10 mL of ultrapure water, 5 mL of EtOH, and 4 mL of MeOH. To remove the dissolved gases, the mixture was ultrasonicated for 5 min and then purged with argon by bubbling. This process also filled the headspace above the liquid with argon, ensuring an inert atmosphere for the synthesis. Subsequently, 2 μL of β-mercaptoethanol was injected to the mixture under vigorous stirring, while the mixture was illuminated with a UV-LED (Thorlabs M365LP1 focused to a 0.5 × 0.5 cm square, 1000 mA driving current, and power density of 500 mW/cm^2^). After 30 min, the mixture was centrifuged at 7000 rcf for 15 min and the precipitate was redispersed in 10 mL of Ar-purged abs. EtOH. After repeating this washing step twice more, the product was redispersed in 1 mL of Ar-purged abs. EtOH and stored in a refrigerator (4 °C, dark).

### Synthesis of Au Nanoparticle-Decorated Cu_2_S Superstructures (Au@Cu_2_S)

The as-synthesized Cu_2_S NPs were dispersed in 10 mL of Ar-purged ultrapure water in a 44 mL sealed glass vial, and the headspace was filled with argon to provide an inert atmosphere. Subsequently, 1 mL of 1 mM aqueous Au^3+^ solution was added to the mixture and then irradiated with a Xe-lamp for 1 h (30 cm distance, 11 mW cm^–2^ power density) under gentle stirring, to deposit gold nanograins on the NPs’ surface. The mixture was then centrifuged at 7000 rcf for 15 min, and the precipitate was redispersed in 10 mL of Ar-purged abs. EtOH. After two additional washing steps, the product was redispersed in 1 mL of Ar-purged abs. EtOH and stored in a refrigerator (4 °C, dark).

### Optical Characterization

UV–vis–NIR extinction spectra of 10-fold diluted ethanolic stock solutions were recorded with a Shimadzu UV3600i spectrometer in UV-transparent disposable semimicro cuvettes (1 cm path length, Brand GmbH.). Absorption spectra in the wavelength range of 250–850 nm were measured with an Edinburgh FS5 spectrofluorometer equipped with an integrating sphere (SC-30 cassette) in QS macro cuvettes.

### Electron Microscopy

Samples for scanning electron microscopy (SEM) and transmission electron microscopy (TEM) were prepared by drop-casting from the freshly synthesized ethanolic stock solutions of the NPs onto Si wafers or carbon-coated TEM grids. SEM images were taken by a Zeiss LEO 1540-XB instrument equipped with an Oxford Instruments UltimMax 40 detector for the EDS measurements. TEM images were recorded at 300 kV in a JEOL 3010 microscope.

### Preparation of Thinned TEM Samples for Phase Identification

Freshly prepared Cu_2_S nanospheres were deposited onto Si wafer (5 × 5 mm) from their ethanolic stock solution and loaded immediately into the focused ion beam (FIB)/SEM (ThermoFischer Scientific Scios2). Thin sections were cut with 30 kV Ga^+^ ions from a selected region covered with several layers of spheres. Further, 5 kV Ga^+^ ions were used to clean the surface. To reach the final thickness and an ultimately clean surface, a low energy (700–300 eV) Ar^+^ ion beam (Technoorg Linda, Gentlemill) was used. The cleaned sample was transferred to a (scanning) transmission electron microscope (ThermoFisher Scientific Titan Themis 200 image corrected with SuperX EDX detectors operated at 200 keV in TEM and STEM modes) immediately to avoid surface oxidation. After recording the selected area electron diffraction (SAED), high-resolution TEM (HRTEM) images, and elemental maps, the evaluation of the SAED pattern was performed by the ProcessDiffraction software.^34–36^

### XRD and XPS Measurements

Freshly prepared ethanolic stocks of the NPs were concentrated by a factor of 20 in an additional centrifuging cycle and then were drop-casted onto precleaned Si wafers (diced, 5 × 5 mm, Ted Pella Inc.). XRD measurements were performed with a Bruker AXS D8 Discover X-ray diffractometer equipped with a CuKα radiation source (λ = 1.5406 Å, beam dimension of 1 × 5 mm), a Göbel-mirror, and a scintillation detector. The 2Θ step size was 0.02°, and the scan speed was 0.2° min^–1^. XPS measurements were carried out on those NP covered silicon wafers that were exposed to air oxygen for the shortest practical time (less than 10 min) of transporting the sample to the vacuum system of XPS. XPS spectra were taken in a ThermoFischer Escalab Xi^+^ (Al Kα radiation source and beam diameter of 0.5 mm) on selected regions with a high NP coverage to suppress the plasmon contribution of the silicon substrate at the sulfur peak position and its oxide layer in the O 1s binding energy range. Spectra were recorded with 0.6 eV energy resolution, 0.1 eV step size, and 0.5 s/point speed, including both XPS peaks and Auger peak of copper. Binding energy scale was adjusted by fixing the adventitious carbon peak at 284.6 eV.

### Preparation of Photoelectrodes

ITO-coated glass slides (VisionTek Systems Ltd., 12–15 Ω/sq) were cut into pieces with dimensions of 5 × 1.5 cm. After cleaning by successive sonication in acetone, 2-propanol, and ultrapure water, the slides were dried with a nitrogen gun. A rectangular mold with an opening of 0.5 × 0.5 cm was prepared from Scotch tape and glued onto the ITO slide. The opening was prewetted with 1 μL ultrapure water followed by the addition of 5 μL (20-fold concentrated) ethanolic stock of the NPs. The droplet was evenly distributed in the mold and dried at ambient conditions, resulting in a homogeneous coating of the particles throughout an electrode surface of 0.25 cm^2^.

### Photoelectrochemical Measurements

Open circuit potentiometry (OCP) and linear sweep voltammetry (LSV) measurements were carried out in a custom photoelectrochemical assembly. Seventeen milliliters of electrolyte (buffer, pH 9) was pipetted into a Hellma Optical Glass cell (30 mm light path, Art. No. 704–001–30–10). A reference electrode (Ag/AgCl in 3 M NaCl, RE-1B, BAS Inc.), a counter electrode (Pt coil, BAS Inc.), and the particle-coated ITO slide as the working electrode were immersed into the electrolyte. The same UV-light source was used to illuminate the particle-coated area of the working electrode as for the synthesis of the Cu_2_S superstructures. However, the current of the LED was set to 1500 mA and the chopping frequency of the source was 50 mHz for LSV (light ON for 10 s, light OFF for 10 s) and 8.3 mHz for OCP (light ON for 60 s, light OFF for 60 s). The potential and current were measured with a PalmSens EMStat4X HR potentiostat/galvanostat. LSV measurements were performed from −1.0 to −0.2 V with a 4 mV/s scan rate. OCP was measured for 800 s with 0.2 s resolution for each sample. The entire setup was placed in a black box to avoid any effect of ambient light.

## Results and Discussion

### Natural Sulfidation of Cuprous Oxide Nanooctahedra

Shape pure Cu_2_O nanooctahedra with average base edge lengths of 161 nm (Figure S1) were synthesized and transferred to an ethanolic medium. Under appropriate storage conditions (in the dark, at 4 °C), the ethanolic stock solution of Cu_2_O nanooctahedra shows outstanding stability: the NPs retain their pristine morphology, with no changes in composition or shape.^33^ However, after prolonged storage exceeding approximately 8 months, small domains began to form at the edges and on the otherwise smooth facets of the NPs, as shown in Figure 1a–d.

**Figure 1:**
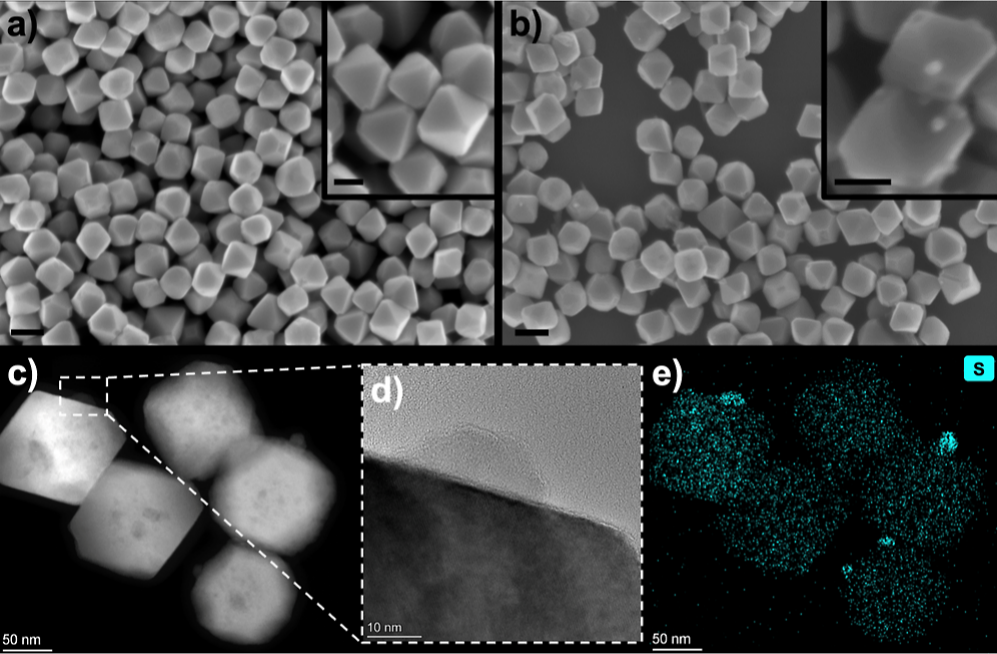
SEM (a,b), TEM images (b,c), and sulfur elemental map (e) of the as-synthesized (a) and aged (b–e) Cu_2_O nanooctahedra. Scale bars are 200 and 100 nm in the SEM images and their insets, respectively.

To shed light on the origin of these protrusions, elemental maps were recorded by STEM–EDX on individual NPs, revealing the presence of sulfur in the surface domains (Figure 1e; elemental maps of Cu and O can be seen in Figure S2). These results suggest that prolonged storage leads to the formation of copper sulfide (Cu_2‑*x*_S) domains on the outer surface of the oxide. Because no sulfur-containing compounds were intentionally introduced to the NP stock solution, this observation can be attributed to the sulfidation initiated by sulfur-containing molecules of ambient atmospheric origin upon short but multiple exposures to air during the sample usage.^37^ To understand and replicate this process, controlled experiments were carried out by systematically introducing a thiol-compound, specifically, β-mercaptoethanol (BME) into the freshly prepared Cu_2_O NP solution.

### Sulfidation of Cu_2_O Nanooctahedra in Dark

BME, as a sulfur source, was meticulously introduced into an as-synthesized Cu_2_O solution. Upon BME addition, small protrusions appeared (Figure 2b) on the crystal facets even for a trace amount (*c*_0_ = 7.15 × 10^–5^ M), resembling those observed during the natural sulfidation caused by the exposure to air (Figure 1). After successfully mimicking a similar transformation in the stock solution, the concentration of BME was increased stepwise to reveal the effect of the short-chain mercaptoalcohol on the structural and optical properties of the NPs (Figure 2). A slight increase in the BME concentration (2–10× *c*_0_) led to the preservation of an original octahedral morphology, however, a gradual increase in the size of the sulfidized domains can be observed (Figure 2b). However, as the amount of the thiol was further increased, the particles’ shape gradually transformed into spherical accompanied by an expansion of the overall particle dimension. Although the octahedral morphology does not change significantly in this concentration regime, surface domains start to evolve. This causes a slight blue shift and broadening in their extinction spectra (Figure 2a), which can be attributed to the increased scattering for Cu_2_O octahedra with surface domains.

**Figure 2:**
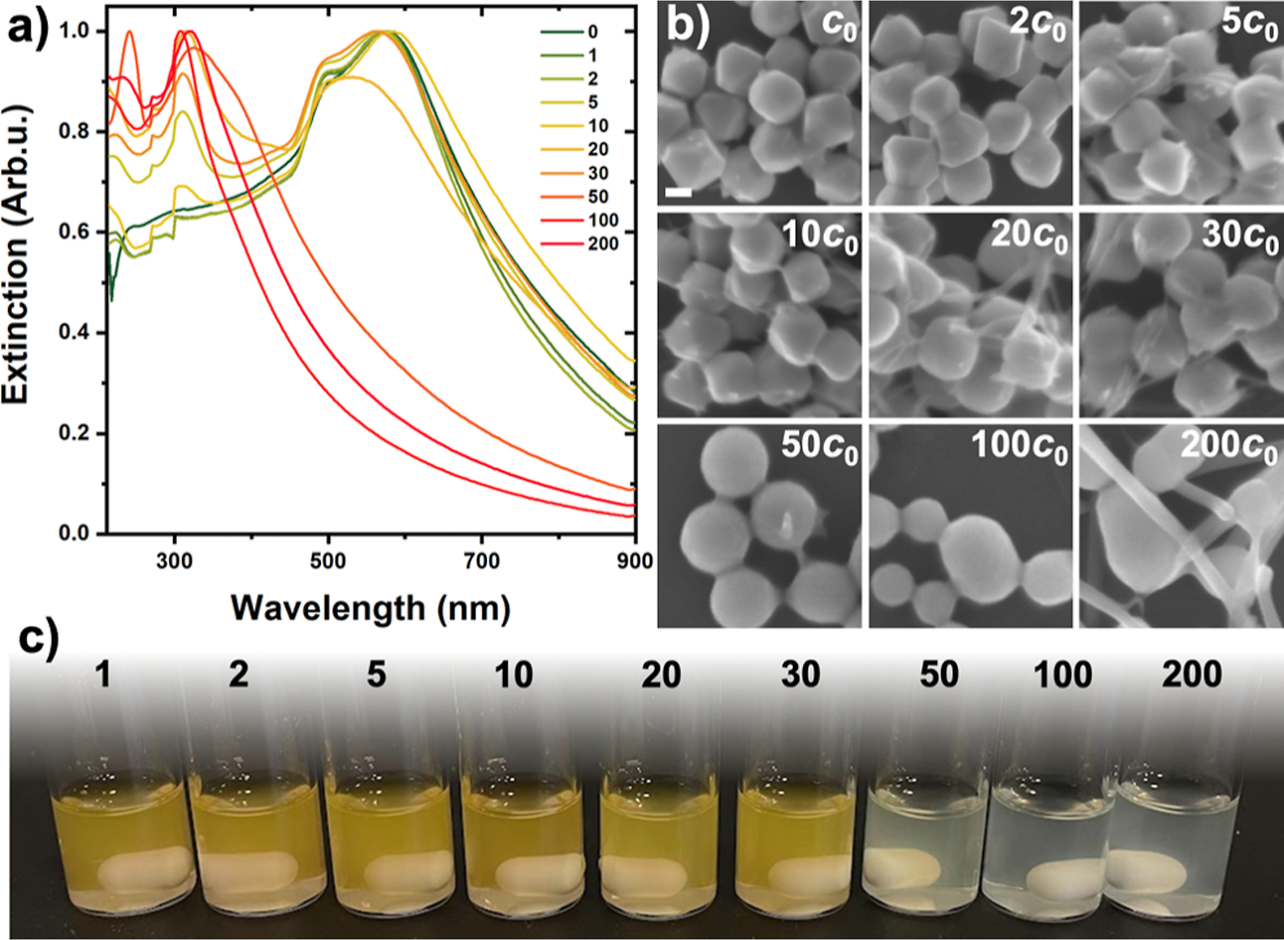
Effect of the BME concentration on the UV–vis spectra (a) and morphology (b) of the NPs, as well as on the color of their dispersion (c). A scale bar represents 100 nm and refers to all SEM images. These experiments were performed in dark conditions.

In addition to the remarkable morphological transformation, a further increase in the BME concentration (20–50× *c*_0_) led to the formation of fibers and the loss of spherical morphology. In this range, the color of the orange NP solution turns white (Figure 2c) and the extinction spectrum changes dramatically. The main extinction peak at ca. 580 nm gradually disappears, and new peaks in the wavelength range of 240–340 nm appear, indicating that the formation of oxidized copper species dominates the optical response and masks the effect of sulfidation. This implies a distinct compositional change in the NPs, which is a superposition of the nanospheres’ material transition as well as the presence of fibers. These fibers also appear in Cu_2_O octahedra if the NP solution is stored in an aqueous medium, where the overoxidation of Cu_2_O to CuO cannot be hindered.^33^ Consequently, controlled sulfidation of cuprous oxide cannot be realized under these conditions due to the overoxidation appearing parallel to the formation of the Cu_2‑*x*_S phase. To address this issue, attempts were made to simultaneously hinder oxidation and promote sulfidation with the aim of preparing phase-pure copper sulfide via sulfidizing the sacrificial Cu_2_O template particles in a controlled manner.

### Photoassisted Sulfidation of Cu_2_O Nanooctahedra

UV light generally facilitates the homolytic cleavage of the thiolic S–H bond generating reactive sulfur species (RSS) in situ. Moreover, charge carriers can be generated in the Cu_2_O octahedra under UV irradiation,^38^ which can make the exposed ⟨111⟩ facets of the particles redox active.^39^ This motivated us to use UV irradiation upon introducing BME to promote RSS formation and, thus, increase the rate of sulfidation over the oxidation process. The two-step synthetic protocol is illustrated in Scheme 1. The process was monitored as a function of the BME concentration by UV–vis–NIR spectroscopy and SEM, and the elemental composition of the formed particles was determined by EDS (Figure 3). As shown in Figure 3c, introducing BME at the lowest concentration (*c*_0_) effectively reproduces what could be observed earlier during the natural sulfidation (Figure 1).

**Scheme 1:**
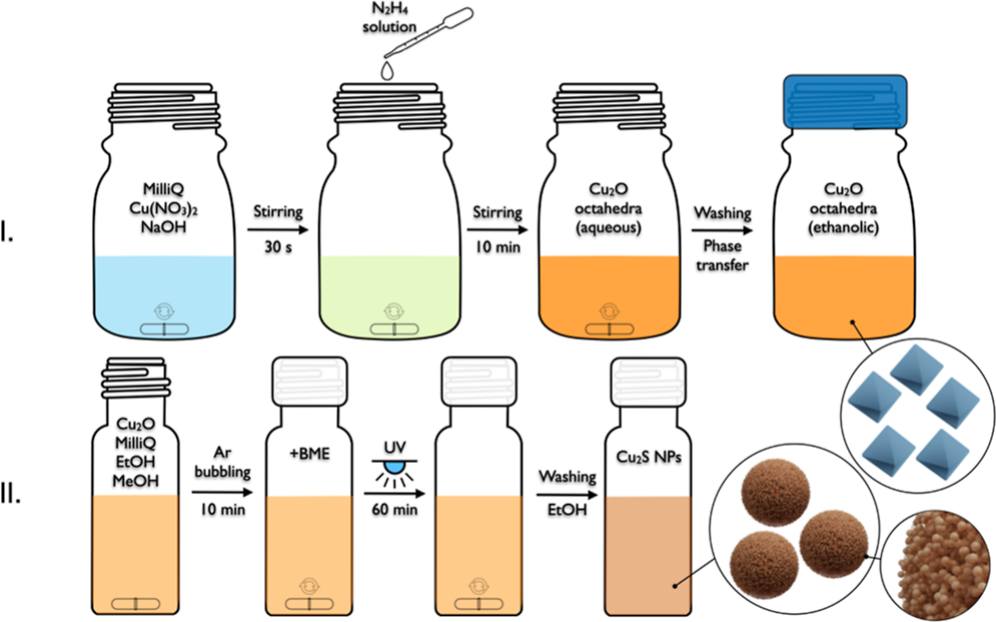
Schematics on the Synthesis Procedure of Cu_2_O Nanooctahedra (I) and the Cu_2_S Quantum-Dot Superstructures (II)

**Figure 3:**
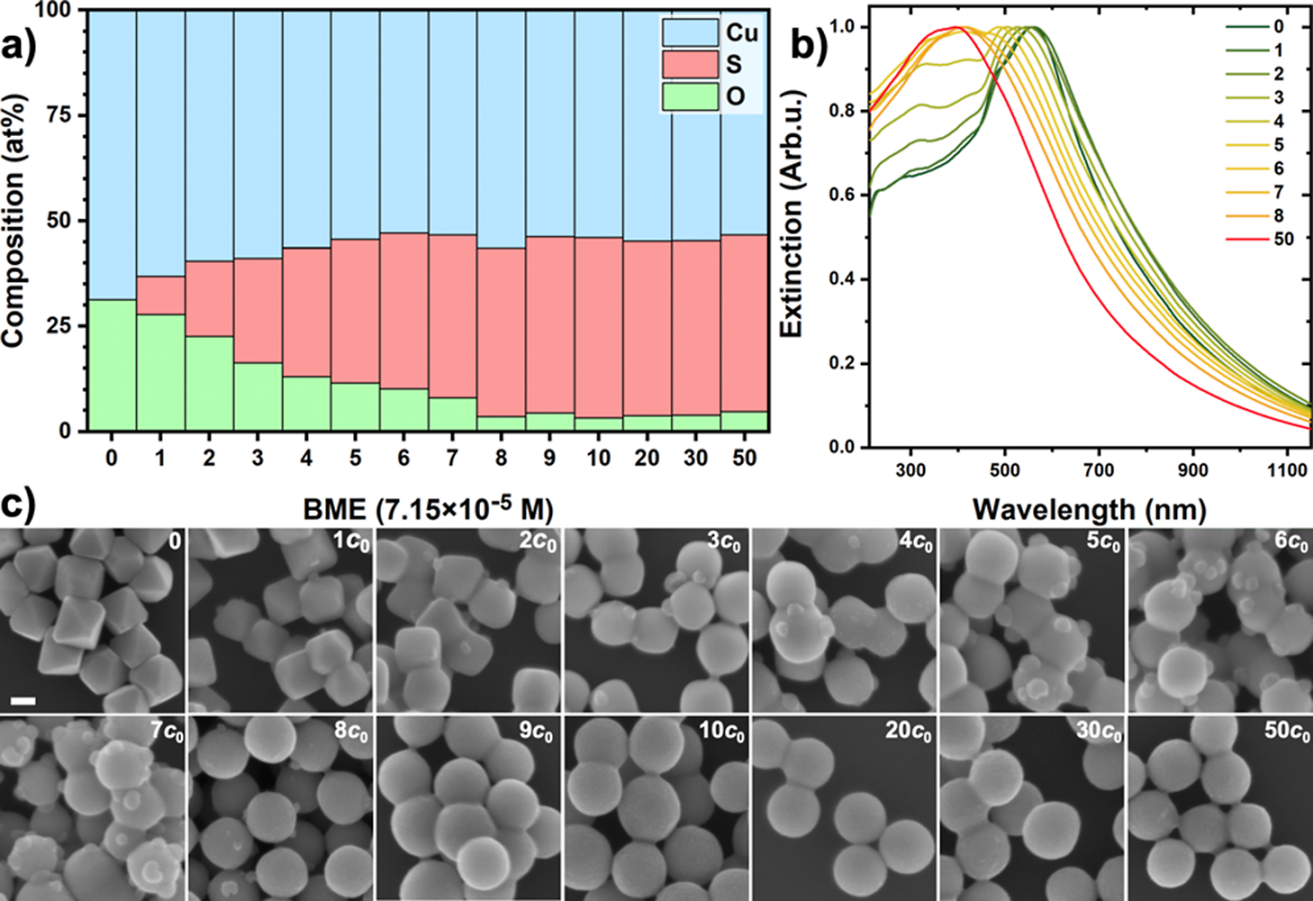
Effect of the BME concentration on the elemental composition (a), UV–vis–NIR spectrum (b), and morphology (c) of the NPs. Scale bar represents 100 nm for all SEM images. These experiments were performed under UV illumination.

With increasing BME concentration, the domains on the particle surface increase, while the octahedral shape gradually turns spherical (Figure 3c, 1–10*c*_0_). Elemental analysis reveals the change in the composition from pure Cu_2_O to Cu_2‑*x*_S with a decreasing oxide content. Further, oxygen atoms can be almost completely replaced by sulfur during this sulfidation process. Once the BME concentration exceeds a certain level (1.43 mM, Figure 3c, 20*c*_0_), the NP morphology and composition change no more. Figure 3b shows that this process is also reflected in the spectral response of the NPs. The increasing BME concentration causes a gradual blue shift and peak broadening in the extinction spectra, while the peak position shifts from 550 to 400 nm. The absence of an emerging peak at 230 nm and the obtained regular nanospheres implies the successful suppression of the UV-induced oxidation during the sulfidation process.

Diminishing the oxidation is further supported by the SEM images showing no fiber-like side products in the entire concentration range of added BME (Figure 3c). The gradual loss of the octahedral symmetry as well as the increasing atomic ratio of S/O suggest a recrystallization of the particles to a Cu_2‑*x*_S phase. To monitor the stability of the sulfidized NPs, their morphology was investigated by SEM over time. When the sulfidation process and the storage of the NP solution were both carried out under ambient conditions, overoxidation of the sulfidized NPs was observed, as shown in Figure S3. However, providing inert conditions by excluding air during both the synthesis and storage enabled the particles to preserve their original morphology for up to 2 weeks, with no signs of overoxidation.

### Structural Properties and Phase Identification of the NPs

Morphology and elemental maps of spherical particles synthesized by the sulfidation of the octahedra under optimized conditions (UV illumination, c_BME_ = 1.43 mM, and alcohol–water mixture) are shown in Figure 4a. Nanospheres with a diameter of about 250 nm are shape-conformed and have a smooth surface and narrow size distribution (Figure S4). EDS mapping reveals a homogeneous distribution of Cu and S, and a low residual oxygen content (ca. 4 at %). More importantly, high-resolution TEM images (Figures 4b and S5) reveal that the nanospheres consist of small, crystalline, and spherical particles in a random orientation. The sizes of these particles are in the range of 4–7 nm, thus, they fall in the size regime of quantum dots.

**Figure 4:**
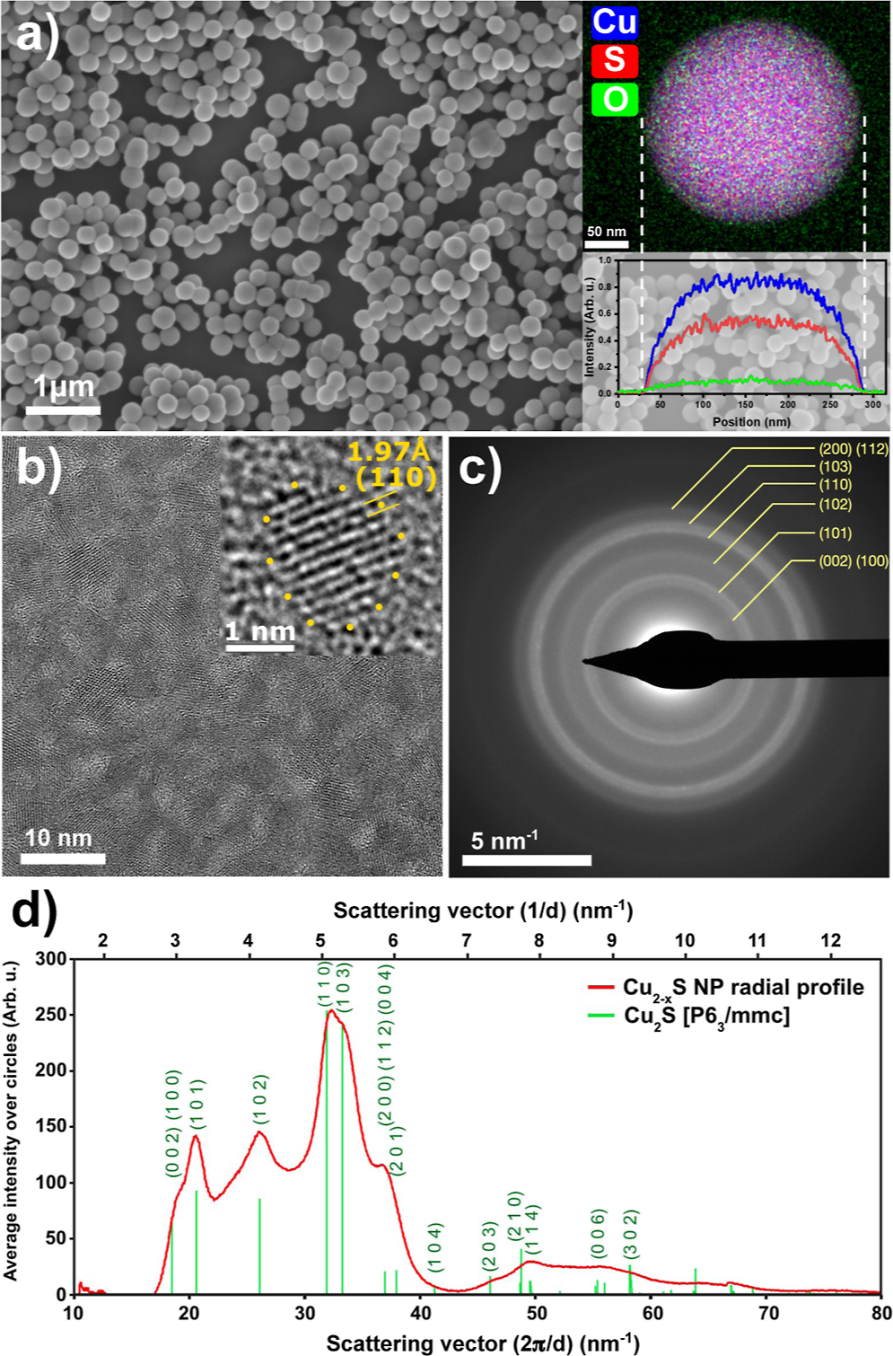
SEM image of the superstructures prepared under the optimal sulfidation conditions (a). Insets show the elemental maps of a single nanosphere and the vertical line profile. HRTEM image (b) of a thinned Cu_2_S particle showing the random orientation of small nanograins as well as an enlarged image of a single quantum dot (inset). SAED pattern (c) of the crystalline domains. Radial average intensity profile of the SAED pattern and the matching diffraction peaks of the hexagonal Cu_2_S phase (d).

To identify the phase of the Cu_2‑*x*_S nanospheres, XRD measurements were performed. While the original Cu_2_O octahedra resemble a cubic crystal structure, diffraction peaks cannot be detected for the sulfidized nanoparticles (Figure S6) likely due to the small size of the crystalline building blocks (quantum dots). Considering the fact that the contribution of sulfur in the EDS measurements can also be attributed to the BME used in the sulfidation process, the elemental distribution itself cannot provide solid evidence of the exact Cu/S atomic ratio in the spheres. Moreover, TEM itself (even at a high resolution) on large nanospheres is not able to provide information about the structural features inside the particles. To address these challenges, an advanced sample preparation technique was employed to identify the phase by means of electron diffraction. This includes the preparation of a TEM lamella by focused ion beam (FIB) followed by its gentle thinning with Ar^+^ ions (see the Experimental Section for details) ensuring that material degradation does not occur during the thinning (Figure S7).

The HRTEM image of the thin section of a nanosphere clearly demonstrates that the building blocks of the nanospheres are crystalline randomly oriented quantum dots with a narrow size distribution throughout the cut segment (Figure 4b). Importantly, selected-area electron diffraction (SAED) on the segment (Figure 4c) can be used to identify the Cu_2‑*x*_S phase in an unambiguous manner. Figure 4d shows the radial profile extracted from the SAED pattern: the position of the diffraction peaks reveals that the phase of the quantum dots is hexagonal (*P*6_3_/*mmc*) Cu_2_S. The identified crystalline phase is pure; other Cu_2‑*x*_S phases or residual Cu_2_O cannot be detected. This implies that the sulfidation of the sacrificial octahedral template results in the formation of superstructures built-up from small phase pure Cu_2_S quantum dots with a homogeneous distribution within the nanospheres (Figure S8).

Figures 4b and S5 also suggest the presence of an amorphous organic material between the Cu_2_S quantum dots. Due to the absence of any organic stabilizer in the synthesis procedure, this can be attributed to the mercaptoethanol attached to the surface of the Cu_2_S QDs within the superstructure. As shown in Figure 3a, the oxygen content in the elemental composition saturates above a certain BME concentration and stays constant at ca. 4 at %. This also implies the presence of BME (as it contains an –OH group) within the superstructure acting as a ligand and a spacer between the QDs.

To verify the oxidation state of the elements, as well as their chemical environment, XPS spectra were recorded. In the binding energy range of copper (2p), peaks at 933.0 (2p_3/2_) and 952.8 eV (2p_1/2_) can be attributed to the Cu_2_S chemical composition without additional oxidation states (Figure 5a). Resolving the XPS spectrum of the S 2p region (Figure 5b) indicates sulfur atoms surrounded by two different chemical environments; the 3/2 and 1/2 doublets at lower binding energy (162.0 and 163.2 eV) correspond to the Cu_2_S, while the same one at higher binding energies (162.7 and 163.9 eV) reveals sulfur connected to carbon. In addition to these two distinct states, a marginal contribution of oxidized sulfur species (SO_*x*_) can also be detected around 167.3 eV without the 3/2 and 1/2 splitting. On the basis of binding energy, the chemical state of oxygen can be linked almost exclusively to the hydroxyl group of the BME (C–O–H bond; Figure S9a). Beside oxygen/sulfur bound carbon, aliphatic C–C bonds can also be detected (Figure S9b). The Auger spectrum of copper (Cu LMM, Figure S9d) also verifies that copper is present in the form of Cu_2_S.^40^ Taking all of these considerations into account, the internal structure of the superstructure can be conceived as Cu_2_S QDs and attached surface ligands (BME). This implies that mercaptoethanol has a dual role; it serves as a sulfidation agent and stabilizes the forming QDs inside the superstructure.

**Figure 5:**
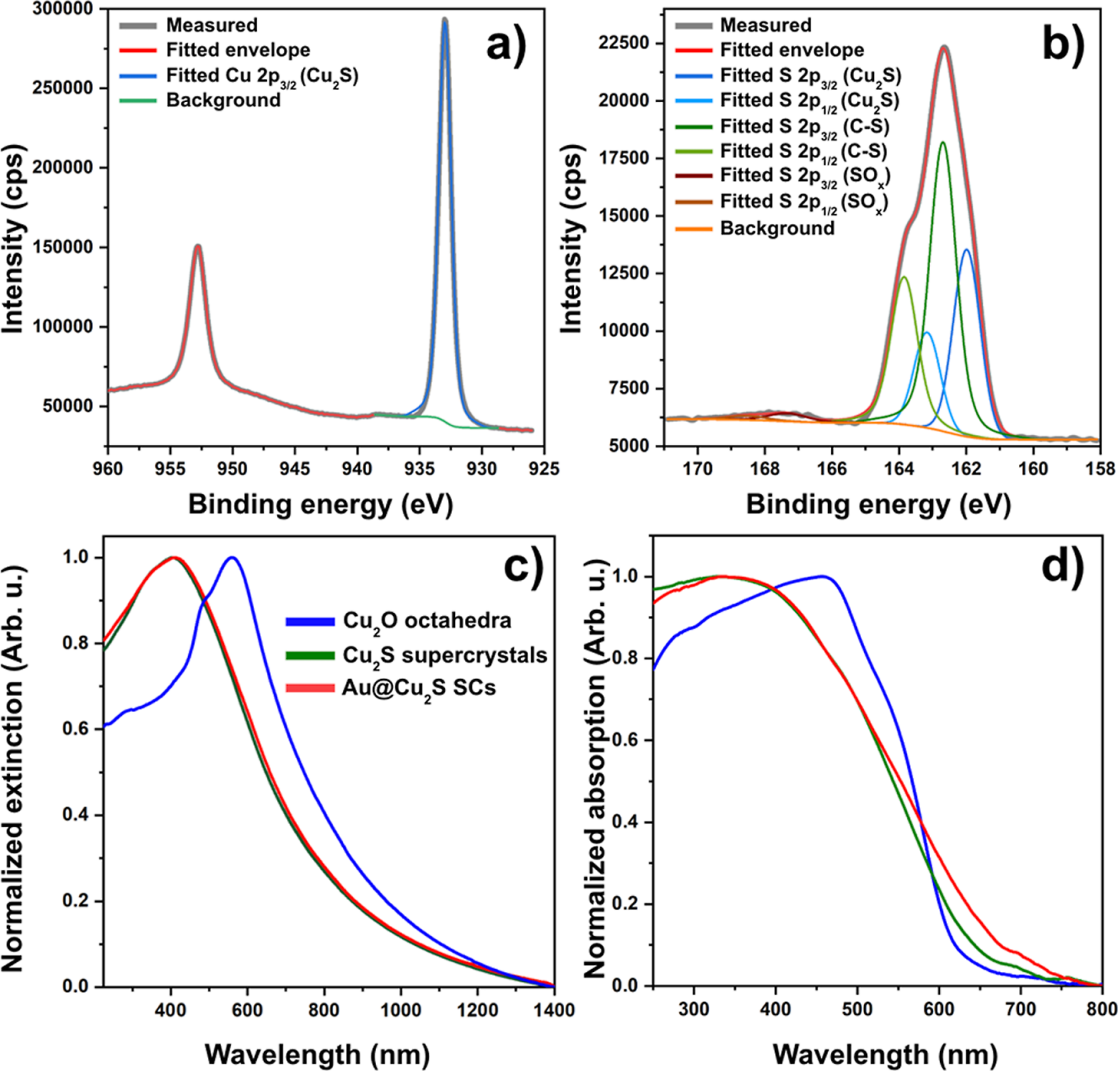
XPS spectra in the binding energy range of Cu 2p (a) and S 2p (b) peaks and the fitted peaks for the Cu_2_S quantum dot superstructures. Normalized extinction (c) and absorption (d) spectra of the model systems synthesized under optimized conditions. Color codes are equal for both panels.

### Decoration of Cu_2_S Superstructures with Au Nanograins

Hybrid semiconductor/metal heteroparticles are able to promote charge-carrier separation processes upon excitation.^41–43^ The metal domain acts as an electron sink which can accumulate photogenerated electrons; thus, the surface decoration of a semiconductor with Au nanograins can enhance the redox capabilities of nanocatalysts.^33^ To endow the QD superstructures with more advanced photofunctional surface properties, Au nanograins were deposited wet-chemically onto the outer surface of the nanospheres (see the Experimental Section for details). To avoid the oxidation of the Cu_2_S surface, the decoration was carried out under an inert atmosphere. Gold is reduced at the surface of the superstructures and forms nanograins with a size of around 4 nm (Figure S10). XRD also proves the presence of crystalline Au domains with a face-centered cubic (fcc) structure (Figure S6).

### Optical- and Photoelectrochemical Properties

Optical properties of sacrificial Cu_2_O octahedra, the Cu_2_S superstructures and Au@Cu_2_S superstructures under optimized synthetic conditions are shown in Figure 5. Cu_2_O octahedra have a distinct extinction peak at 560 nm (Figure 5c), with a slight shoulder at 490 nm. The former is the resonant Mie scattering of the particles, while the latter can be attributed to the band-to-band transition. Upon sulfidation, the peak significantly blue-shifts (peak centers at 400 nm) and slightly broadens. This peak can be attributed to the band-edge absorption of the QDs having size-quantized optical properties,^44^ while the broad extinction at higher wavelengths is related to the scattering of the superstructures. Similarly, the band-edge absorption was found earlier at 410 nm for dispersed aqueous Cu_2_S QDs,^45^ and the observed peak position is in-line with the reported value for polyhedral Cu_2_S nanoparticles with similar sizes.^46^

Decoration of the surface with Au nanograins does not alter the extinction spectrum significantly: scattering masks the contribution of adventitious extinction of the small Au nanoparticles. Optical measurements in an integrated sphere aid the separation of the scattering and the absorption. Figure 5d shows the pure absorption of the nanoparticle solutions. The band-edge absorption is located at 480 nm for Cu_2_O nanooctahedra, and at 400 nm for Cu_2_S and Au@Cu_2_O particles. The deposition of gold causes a slight broadening above 550 nm; however, it does not modify the band-edge absorption of the Cu_2_S quantum-dot superstructures. Extinction and absorption measurements imply that despite the large scattering superstructures, the optical properties are dictated by the confined semiconductor QDs.

Being Cu_2_S a semiconductor, the investigation of the photoelectrochemical properties is essential to gain insights into photoinduced processes. Thus, working electrodes were prepared by drop-casting the concentrated nanoparticle solutions onto a molded ITO-coated glass substrate, and an electrochemical setup was assembled (details of electrode preparation and the setup can be found in the Experimental Section). The nature of the semiconductor was determined by open circuit potentiometry, where the change in the equilibrium potential of the photoelectrode was monitored in time upon chopped UV-light irradiation. As shown in Figure 6a, irradiation induces a negative shift in the open circuit photovoltage (that is more negative OCP) for ITO-coated glass as expected for the n-type ITO.^47^ Note that the photoinduced response of ITO is sluggish, and the equilibrium is not reached during the alternating on/off periods. In contrast, all the three synthesized model systems (Cu_2_O, Cu_2_S, and Au@Cu_2_S) show an opposite response (positive shift) as a result of the p-type nature of both Cu_2_O and Cu_2_S and implies that the surface of the working electrode accumulates positive charges (holes).^48^ Upon UV irradiation, the response is prompt, and the potential reaches an equilibrium in irradiated as well as in dark phases.

**Figure 6:**
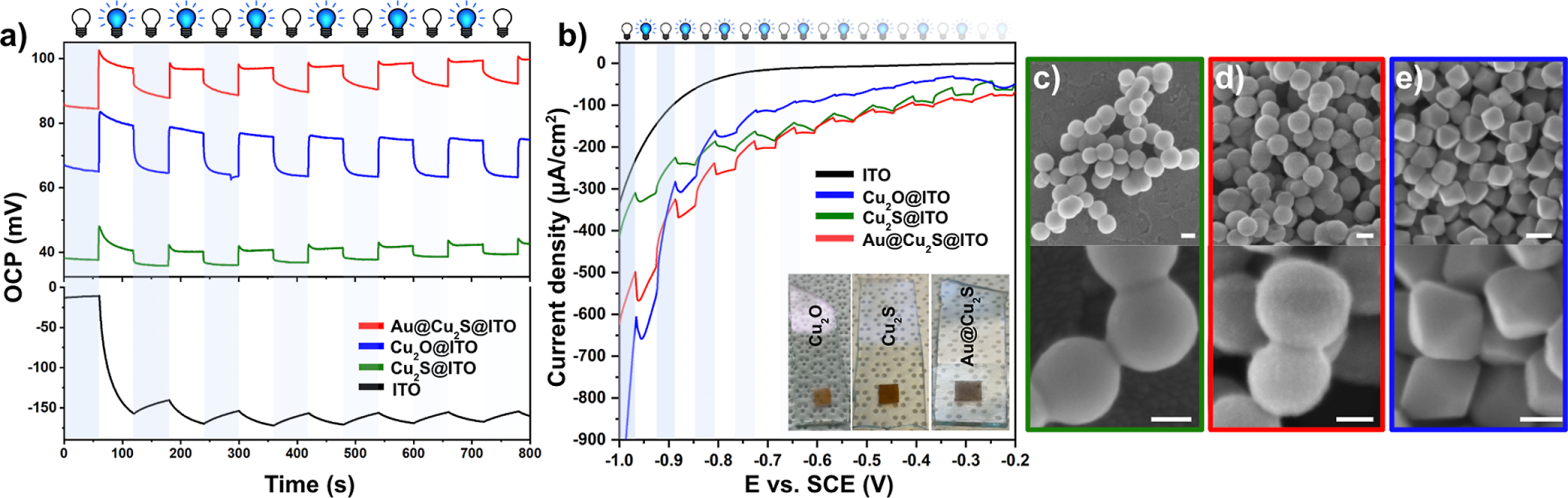
Open circuit photovoltage as a function of time for the investigated model systems (a). Linear sweep voltammograms in the negative bias potential range under anodic polarization (b). SEM images of the Cu_2_S (c), Au@Cu_2_S (d) nanospheres, and Cu_2_O nanooctahedra (e) after the LSV measurement. White and blue bulbs represent the OFF and ON states of the UV illumination source, respectively. Insets of panel b show the physical appearance of the working electrodes. Scale bars for the SEM images are 200 nm (upper row) and 100 nm (bottom row).

Deposition of gold onto the superstructures slightly increases the photovoltage under irradiation compared to the Cu_2_S nanospheres; however, it does not change their p-type character. The observed retardation in the equilibration of the OCP during dark periods, when Au NPs are present, indicates enhanced charge separation at the Cu_2_S/Au NP interface. This phenomenon is attributed to the presence of an internal electric field facilitating the separation of photoexcited charges. To further elucidate the photoelectrochemical response of the nanoparticle-coated electrodes, linear sweep voltammetry under chopped UV light irradiation was performed. Considering the fact that the applied potential governs the redox reactions at the photoelectrode–electrolyte interface, negative potentials were applied to avoid the photocorrosion^49^ (see Figure S11 for the effect of positive polarization). During the anodic polarization of the supporting ITO in alkaline pH, the photogenerated cathodic current cannot be detected (Figure 6b). On the contrary, a negative photocurrent occurs in the presence of copper-based semiconductor nanoparticles. The Cu_2_S and Au@Cu_2_S superstructures show a cathodic photoresponse in the entire negative bias range surpassing the photocurrent of Cu_2_O nanooctahedra in the range from −0.9 V to −0.2 V. For Au@Cu_2_S particles, at −0.8 V, the photocurrent reaches the value of 50 μA/cm^2^ (referring to the dark vs irradiated state), which is significantly higher compared to the one for multifaceted Cu_2_S particles.^46^ Furthermore, an enhanced cathodic photocurrent can be generated upon decorating the Cu_2_S superstructures with Au nanograins. When Au nanoparticles are brought into contact with Cu_2_S, a Schottky junction is created that facilitates the accumulation of holes at the surface of Cu_2_S in equilibrium. The Schottky junction at the Au/Cu_2_S interface creates an internal electric field that aids the separation of the photogenerated electron–hole pairs. Under a cathodic bias, this field is enhanced, directing photogenerated electrons more efficiently to the surface, reducing recombination upon transferring to the electrolyte. Moreover, the cocatalytic effect of AuNPs cannot be excluded from the contributing effects.

Below −0.85 V, Cu_2_O nanooctahedra supply a higher photocurrent compared to the Cu_2_S and Au@Cu_2_S superstructures, which can be attributed to the differences in the density of the electrochemically reducible states. The contribution of these states is likely attenuated upon an initiation period at −1.0 V in the case of the superstructures.

Notably, the irradiated nanoparticles retain their morphology, and no signs of surface degradation can be observed upon cathodic polarization during the LSV experiment, as evidenced by follow-up SEM analysis (Figure 6c–e).

### On the Mechanism of the Sulfidation Process

Using BME as a sulfidation agent in the same concentration in the dark and under light irradiation results in strikingly different evolution of the particles. Furthermore, solution-based chemical transformations are highly dependent on the properties of the solvent, which govern factors such as solubility, ion mobility, and the surface characteristics of the nanoparticles following selective adsorption. While the optimal sulfidation of the octahedra takes place in alcohol–water solvent mixture, BME in the same concentration cannot entirely sulfidize the Cu_2_O octahedra in an ethanolic medium. Without water, the sulfidation process is spatially limited and leads to the formation of core–shell Cu_2_O/Cu_2_S nanooctahedra (Figure S12). Additionally, the exclusion of alcohols in the synthetic procedure leads to the formation of coalescing nanospheres (Figure S12a), which also indicates that the ion mobility is of crucial importance in the process: while pure aqueous medium accelerates, pure ethanolic medium slows the sulfidation reaction considerably.

The formation of core/shell particles with an octahedral morphology in abs. ethanol implies that the sulfidation begins at the surface of the octahedra, and it is likely not a dissolution/recrystallization reaction. In contrast, in the presence of water, sulfur species might diffuse into the copper-oxide lattice and promote anion-exchange in the sublattices. These solvent-dependent morphological changes pin down the importance of the ion mobility during the transformation.

Upon the formation of Cu_2_S quantum dots, however, the small grain size might limit the stability; thus, the surface attachment of mercaptoethanol is required to reduce the surface energy. It is important to emphasize that Cu_2_S quantum dots form phase pure crystallites that do not contain BME in their lattice. This approach differs fundamentally from the use of thiolated molecules (e.g., glutathione) to form amorphous copper-based superstructures without a crystalline Cu_2‑*x*_S phase as, for example, reported by Dai el al.^50^ In the current case, the reactive sulfur species (RSS), which are able to replace oxygen to sulfur in the sublattice, are more likely in situ generated from the thiol. In the sublattice of the ⟨111⟩ facet of Cu_2_O, O can be replaced by S as DFT calculations indicated.^51^

The formation of reactive species can be facilitated by high-energy UV irradiation either by the homolytic cleavage of the S–H bond (having low bond dissociation energy)^29,52^ or the cleavage of the disulfide bond (RS-SR) initiated by the UV light.^53^ Thiols and the so-formed thiolates and thiyl radicals can strongly interact with the copper centers at the surface of Cu_2_O.^29^ In parallel to the generation of RSS, UV-light can generate charge carriers in the semiconductor itself. In the presence of oxygen, photogenerated holes can oxidize Cu_2_O to CuO, which manifests as overoxidation of the particle surface. Photogenerated electrons, on the other hand, can promote the reduction of the surface-bound Cu-SR complexes, providing the sulfur species necessary for Cu_2_S formation. A similar mechanism with biothiols has been reported on Au@Cu_2_O particles: the presence of cysteine and glutathione triggered the dissolution of the Cu_2_O shell via strong Cu–S bond formation, while the presence of CuO could also be detected.^54^ Under an inert atmosphere, the oxidation of Cu_2_O can be readily avoided, thereby favoring sulfidation over oxidation. In our case, unbound S^2–^ species cannot be detected via XPS, and thus, the in situ formation of the species governing the anion-exchange reaction upon UV irradiation can be anticipated.

## Conclusions

Environmental sulfidation of Cu_2_O nanoparticles can occur at ambient conditions, that is, upon exposing the NPs to the atmosphere. Following a certain storage period, copper sulfide domains emerge at the surface of Cu_2_O nanooctahedra, even without introducing sulfur compounds. Inspired by this natural phenomenon, we have triggered the direct sulfidation of Cu_2_O nanoparticles by short-chain mercaptoethanol wet-chemically to form Cu_2‑*x*_S particles. The resulting structure and composition during this transition, however, depends heavily on the conditions of the sulfidation. In dark conditions, the introduction of BME induces the partial sulfidation as well as the oxidation of the nanooctahedra at ambient conditions. The suppression of overoxidation and realization of phase-pure Cu_2_S nanoparticles can be achieved by the photoassisted sulfidation of Cu_2_O template particles in an inert alcohol–water solvent mixture. Under optimal conditions, direct sulfidation ensures the preparation of spherical Cu_2_S nanoparticles composed of small Cu_2_S quantum dots with attached mercaptoethanol ligands on their surface. A semiconductor–metal multicomponent heterostructure can also be synthesized by decorating the surface of Cu_2_S superstructures with Au domains in a photoreduction pathway. Fabricating working electrodes by depositing Cu_2_S and Au@Cu_2_S particles on a conductive substrate enables the investigation of the photoelectrochemical properties of the novel model systems. An open circuit photovoltage and chopped-light linear sweep voltammetry measurements confirm the p-type nature of the particles and the evolution of negative photocurrent is achieved, which is further improved in the presence of Au nanograins. Phase-pure Cu_2_S quantum-dot superstructures can serve as novel nanostructured materials with photofunctional properties, controlled composition, and controlled morphology.

## Supplementary Material



## Notes

SEM and TEM images of the Cu_2_O nanooctahedra and Cu_2_S superstructures; HRTEM images of the thinned sections; XRD patterns; additional HAADF image and elemental maps; XPS and Auger spectra; SEM, TEM images, and elemental maps of Au@Cu_2_S particles; additional LSV measurements; SEM, TEM images, and elemental maps of Cu_2_O/Cu_2_S core/shell particles; and stability of the particles over time (PDF)

## Abbreviations

NP: nanoparticle
BME: β-mercaptoethanol
SEM: scanning electron microscopy
FIB: focused ion beam
TEM: transmission electron microscopy
EDS: energy-dispersive X-ray spectroscopy
SAED: selected-area electron diffraction
XPS: X-ray photoelectron spectroscopy
OCP: open circuit photovoltage
LSV: linear sweep voltammetry
QD: quantum dot.
