# Supplementary material for: Photoassisted Chemical Transformation of Cu2O Nanooctahedra into Cu2S Quantum-Dot Superstructures: Structural and Photoelectrochemical Properties
Source: ACS Mater Au. 2025 Aug 13;5(6):1018–28. doi: 10.1021/acsmaterialsau.5c00106 (PMC12616430; doi:10.1021/acsmaterialsau.5c00106)
Supplement: Supplementary file 1 [file mg5c00106_si_001.pdf]

Supplementary Information

Photoassisted chemical transformation of Cu_2O nanooctahedra to Cu_2S quantum-dot superstructures: structural and photoelectrochemical properties

Dávid Kovács,[a,c] György Z. Radnóczi,[a] Zsolt E. Horváth,[a] Krisztina Frey,[b] Attila Sulyok,[a] Zsolt Fogarassy,[a] József S. Pap,[b] András Deák,[a] Dániel Zámbó[a]*

[a]HUN-REN Centre for Energy Research, Institute of Technical Physics and Materials Science, Konkoly-Thege M. út 29-33., H-1121 Budapest, Hungary

[b]HUN-REN Centre for Energy Research, Institute of Energy Security and Environmental Safety, Konkoly-Thege M. út 29-33., H-1121 Budapest, Hungary

[c]Budapest University of Technology and Economics, Department of Physical Chemistry and Materials Science, Műegyetem rkp. 3., H-1111 Budapest, Hungary

*e-mail: daniel.zambo@ek.hun-ren.hu

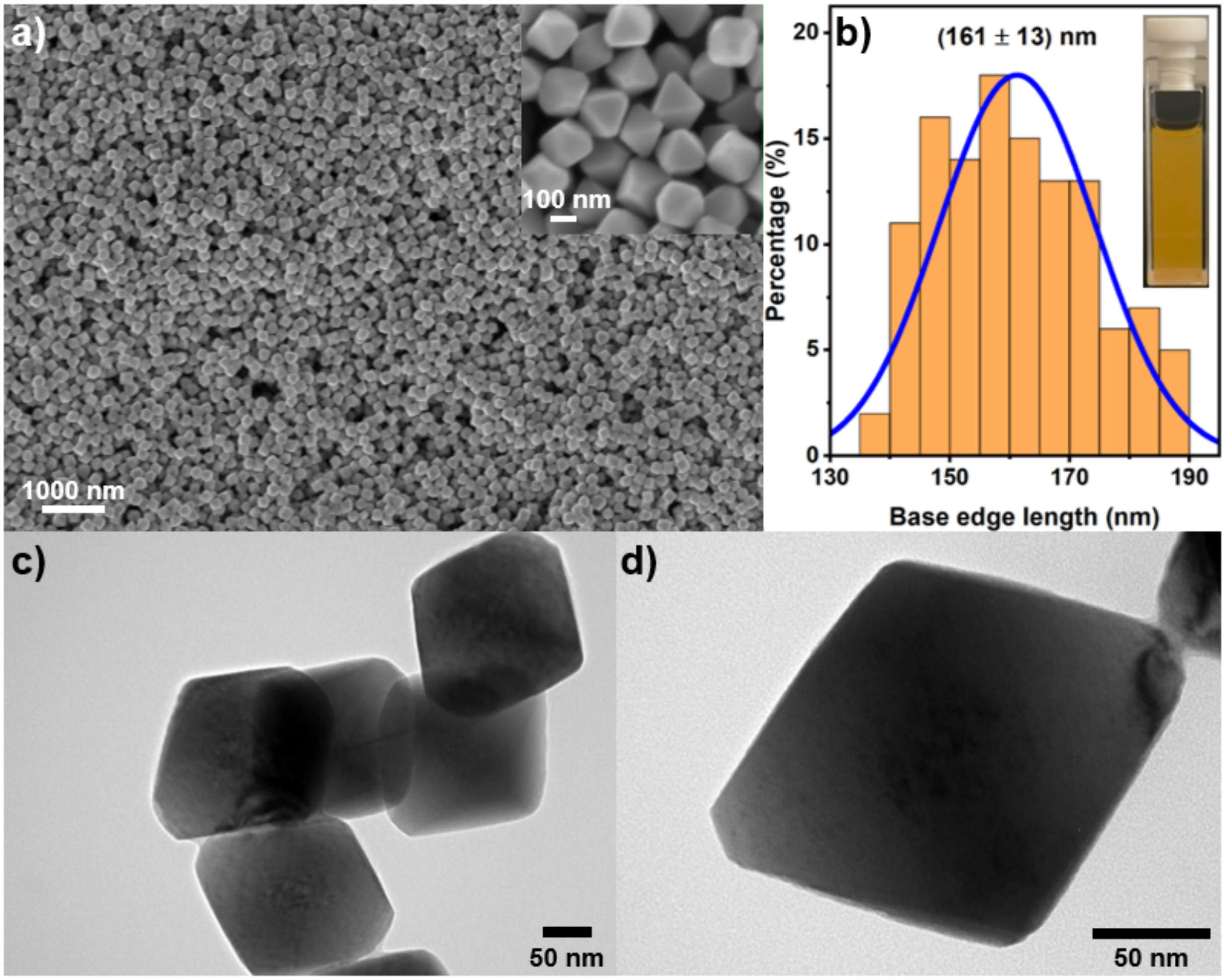

Figure S1. SEM (a), TEM (c,d) images and base edge length distribution (b) of the synthesized Cu_2O nanooctahedra used for the sulfidation experiments.

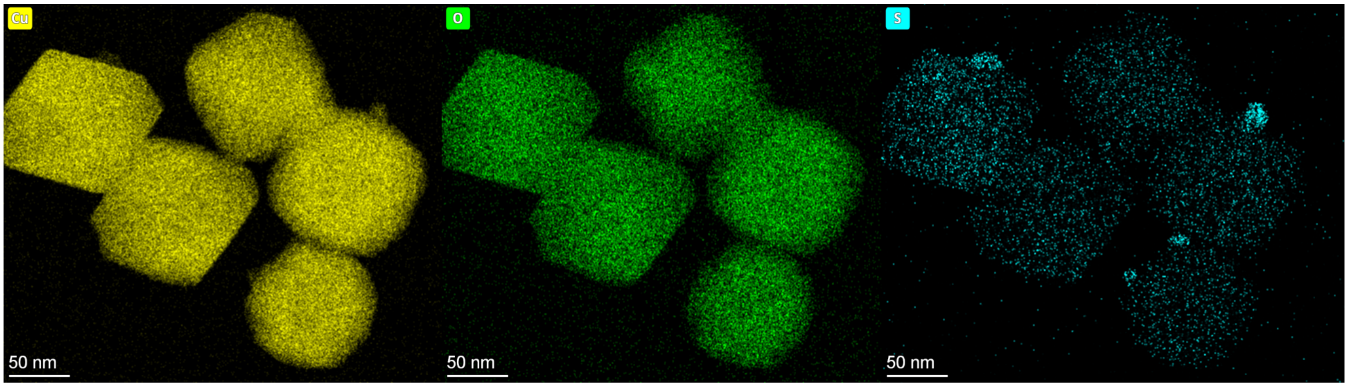

Figure S2. Elemental maps (Cu, O, and S) of the naturally sulfidized Cu_2O octahedra.

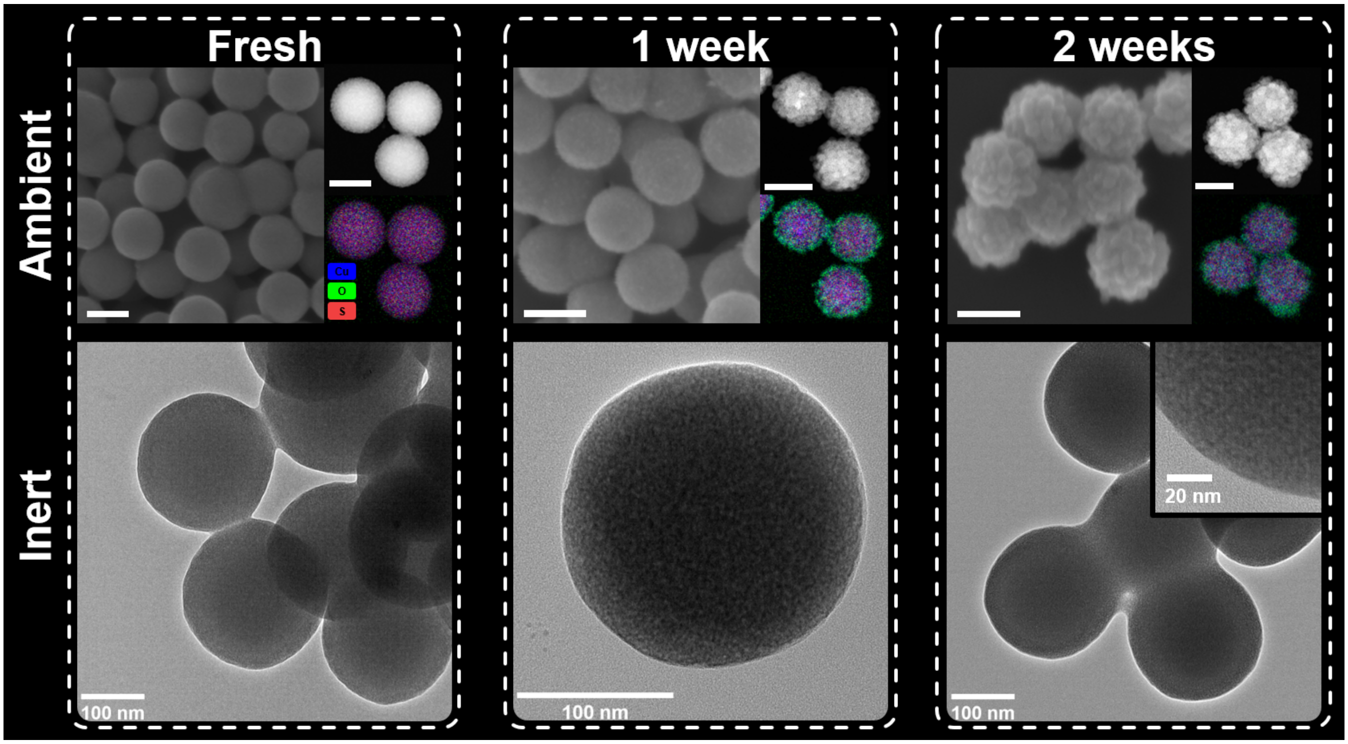

Figure S3. Comparison of ethanolic NP stock solution stability when the particles are synthesized and stored under ambient or inert conditions. In the ambient case, the originally smooth-surfaced particles begin to oxidize within a week that becomes more severe over time, which is confirmed by TEM-EDS elemental mapping. In the inert case, the NP surfaces show no signs of overoxidation, even after two weeks. Scale bars in the SEM images and elemental maps represent 200 nm.

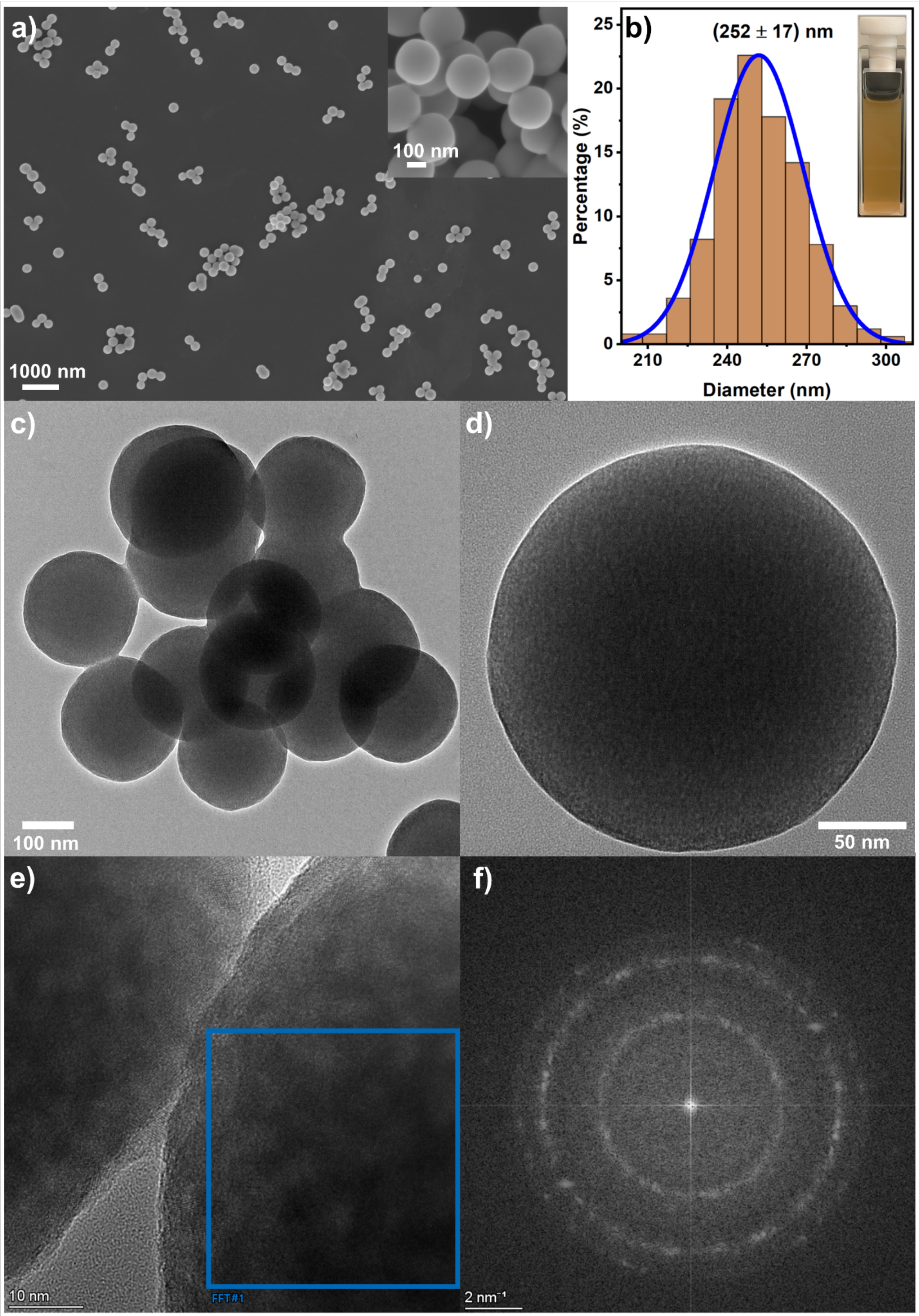

Figure S4. SEM (a), TEM (c,d) images and size distribution (b) of the synthesized Cu_2S particles. HRTEM image (e) and FFT of the area marked by the blue square in the HRTEM image (f).

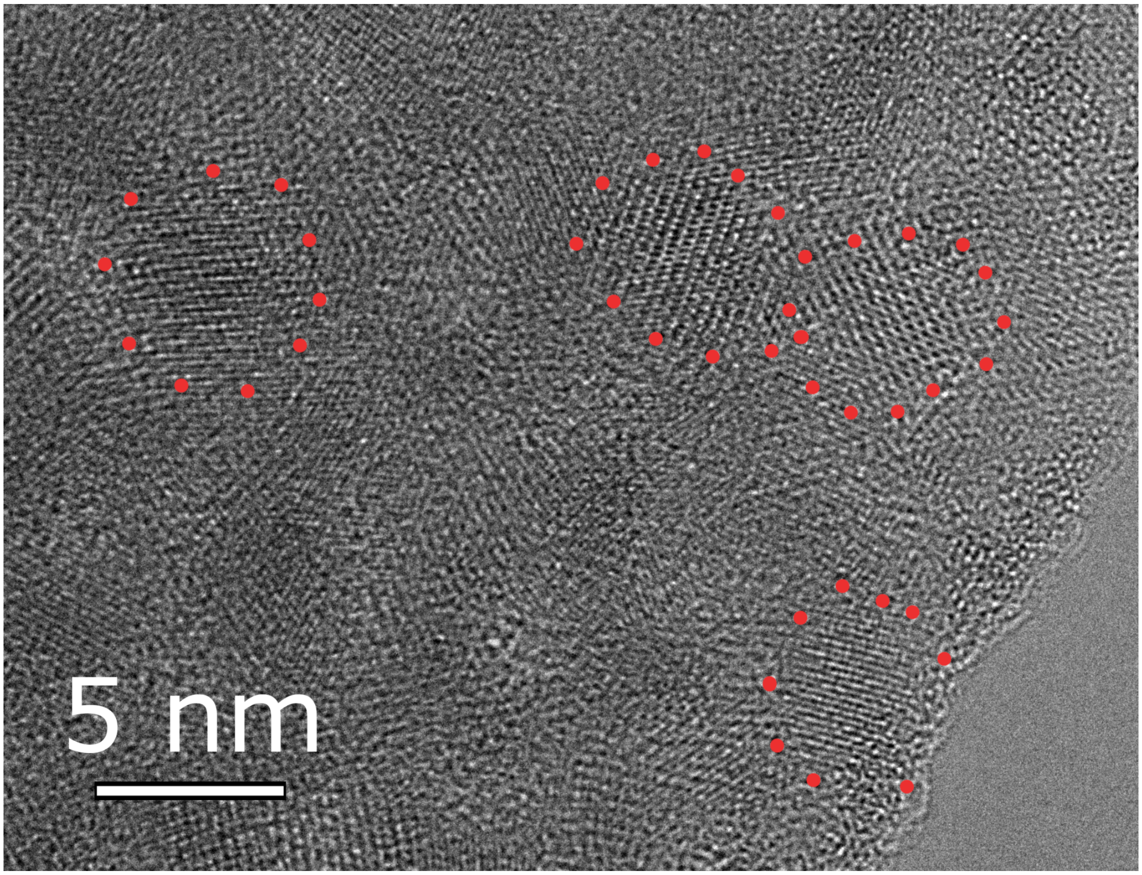

Figure S5. HRTEM image of a thinned region of a Cu_2S superstructure. Crystalline grains which exhibit lattice fringes clear enough for size estimation are highlighted by red dots as guide to the eye. The average size of the grains is in the range of 4-7 nm.

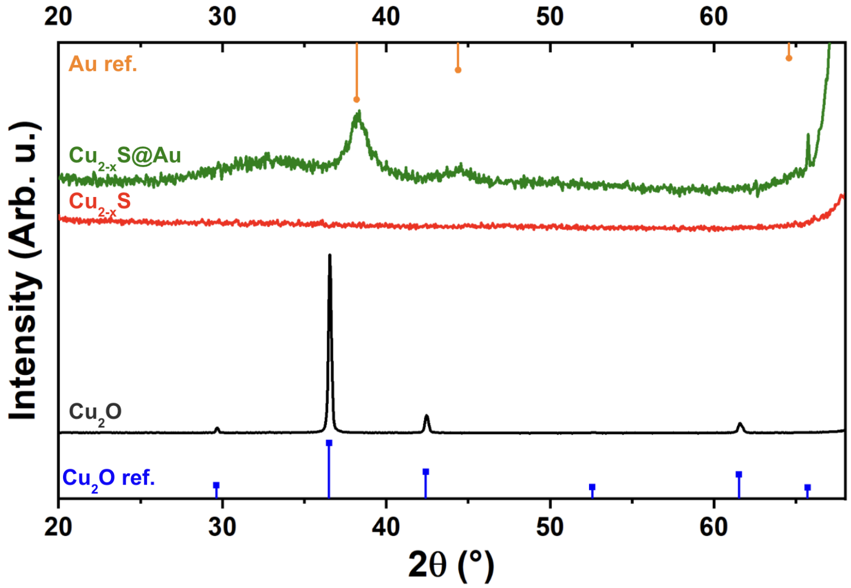

Figure S6. XRD pattern of the synthesized particles and the corresponding reference lines of Cu_2O (PDF 01-077-0199) and Au (PDF 03-065-2870). It is visible that Cu_2S was not detected in the superstructures due to the small grains size and their random orientation.

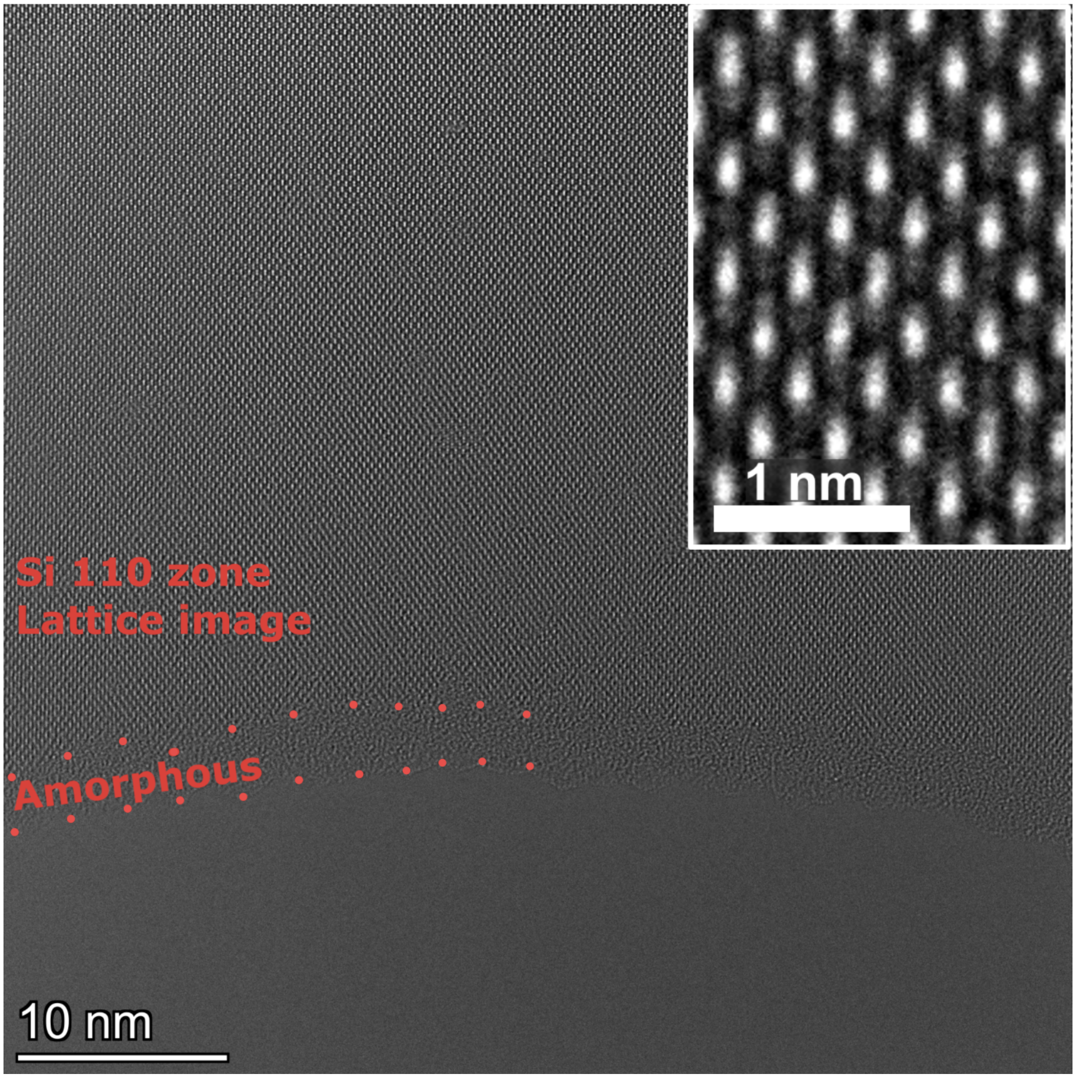

Figure S7. HRTEM image of the Si substrate showing the result of the low energy Ar^{+} ion beam cleaning. A 3 nm wide amorphous band appears at the lower edge of the Si crystal which is attributed to the native oxide formed on the surface after cleaning. Further away from the edge (but still in very thin regions) the specimen exhibits a good quality lattice image (inset) indicating a clean and undamaged surface.

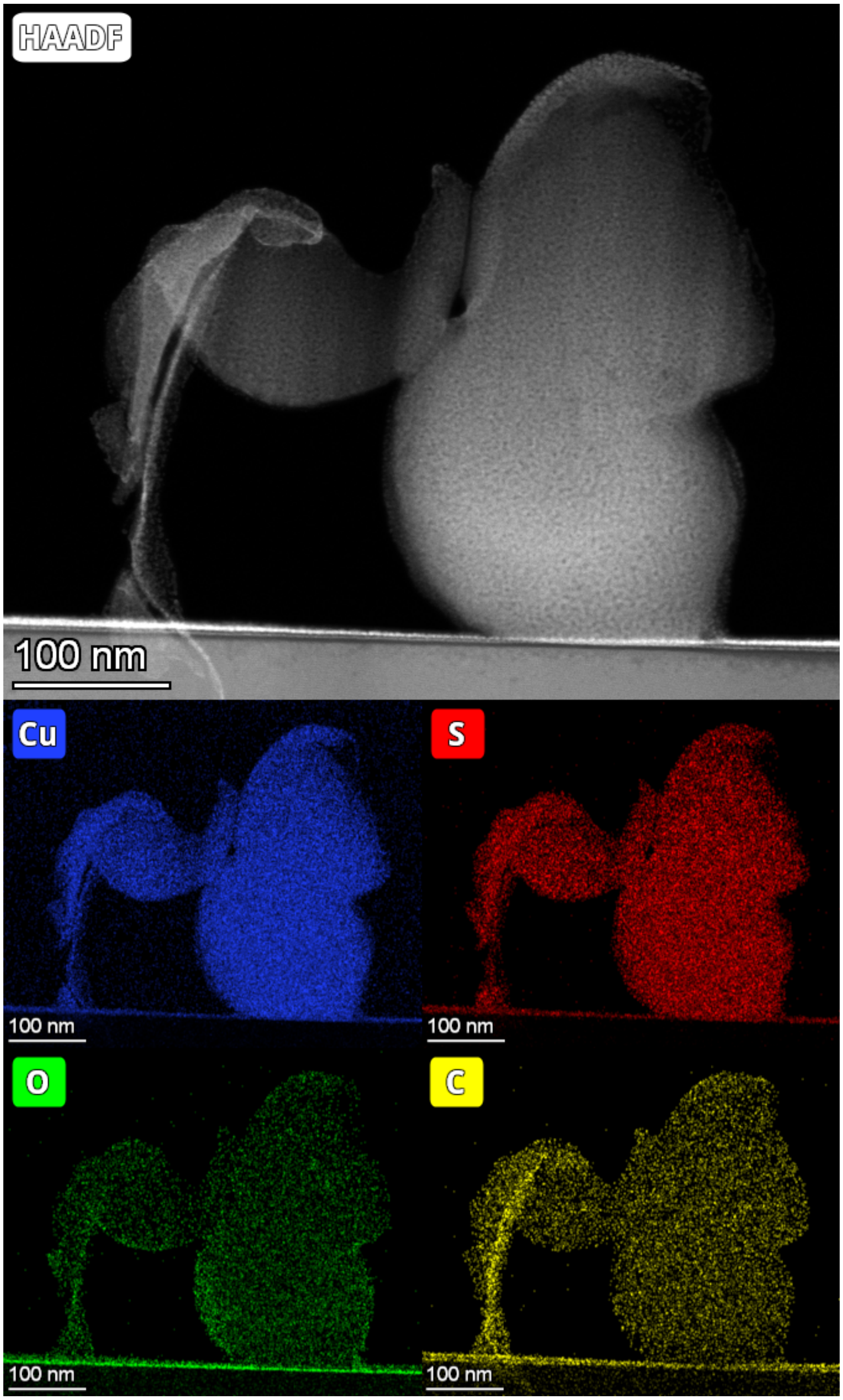

Figure S8. HAADF image and elemental maps of a thin section prepared from the Cu_2S superstructures. Elemental maps (at%) for Cu, S, O and C show a homogeneous distribution of elements.

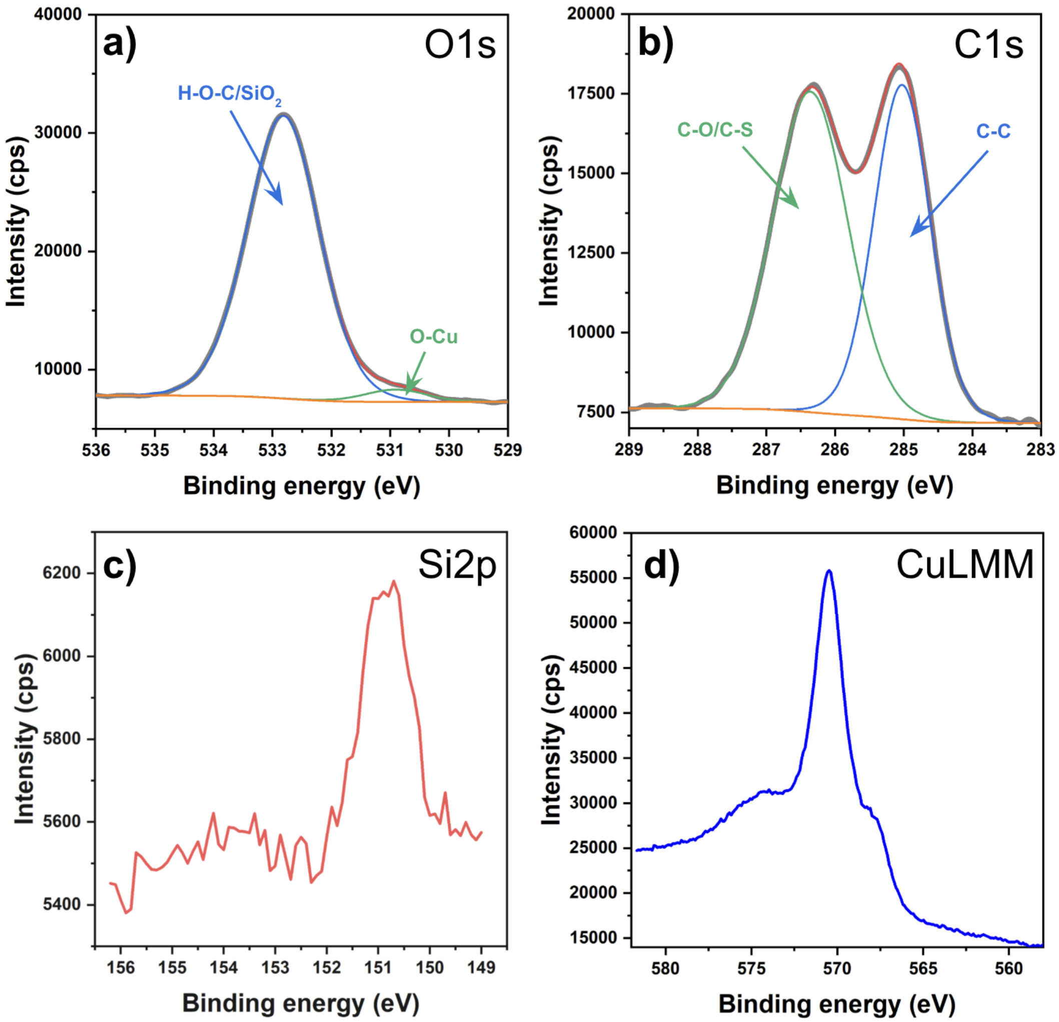

Figure S9. XPS (a-c) and Auger spectrum (d) of oxygen (a), carbon (b), silicon (c) and copper (d) in Cu_2S superstructures. In the XPS spectra (a,b), dark grey, red and orange curves represent the measured, fitted envelope and background, respectively. Low intensity in the Si2p spectrum shows that the measured area of the sample was almost entirely coated with the Cu_2S superstructures, thus, contribution of amorphous SiO_x at the silicon surface is negligible in the O1s spectrum and the main O1s peak at 532.7 eV can be attributed to the presence of hydroxyl groups.

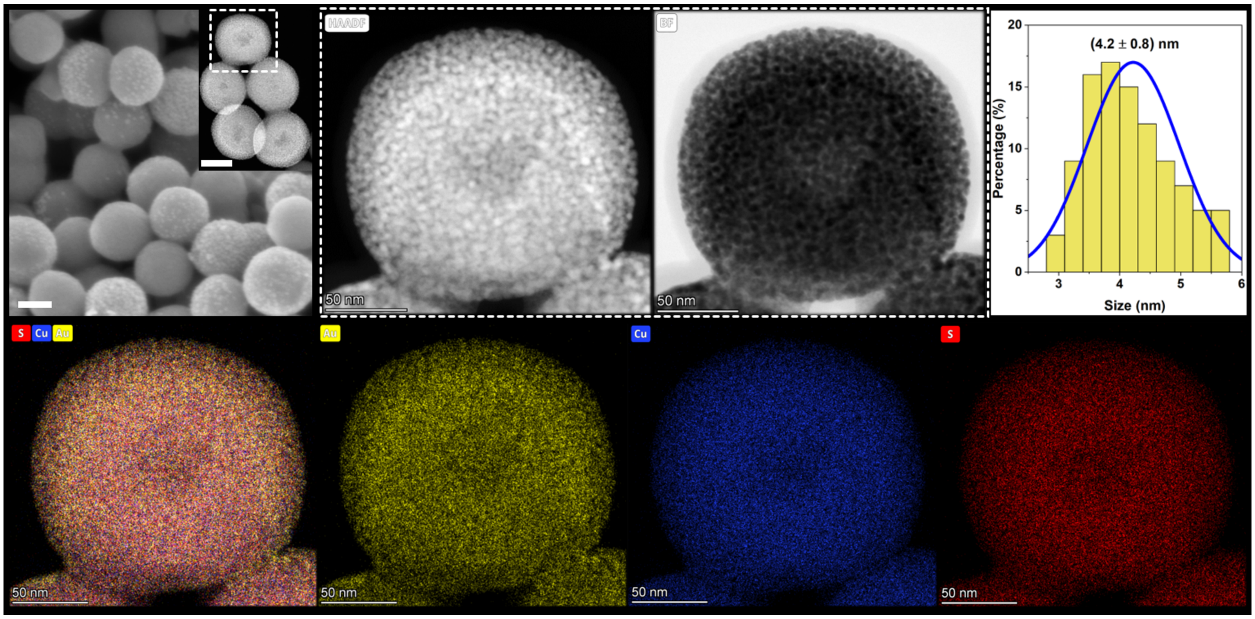

Figure S10. SEM, TEM (HAADF, BF) images and elemental maps of Au NP decorated Cu_2S superstructures (Au@Cu_2S), as well as the size distribution of the Au nanograins. Scale bars in the SEM image and in its inset represent 100 nm.

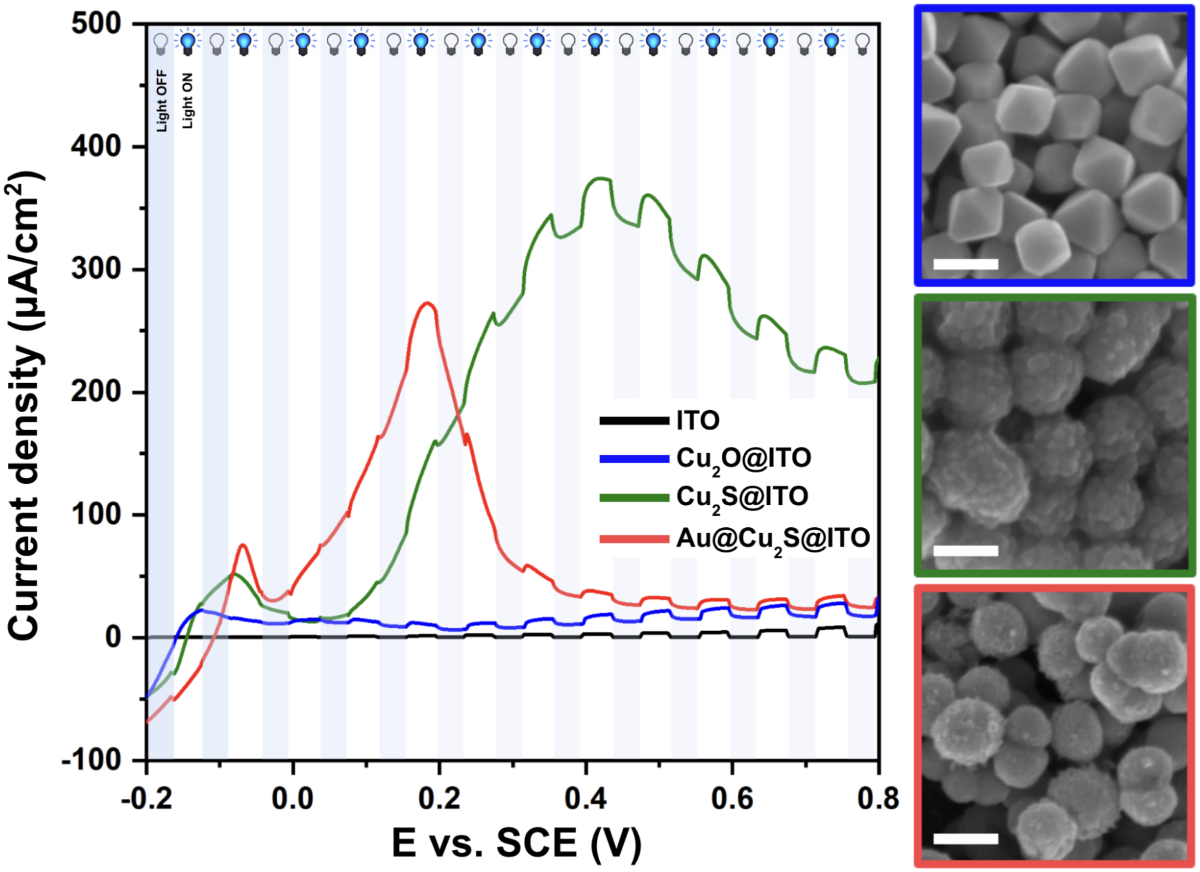

Figure S11. LSV measurements under chopped UV-light irradiation and anodic polarization. SEM images of the NPs after the LSV measurements. Scale bars represent 200 nm in all SEM images. Oxidation peaks for Cu_2S and Au@Cu_2S samples indicate the formation of copper-oxide which is also reflected in the morphological changes shown in the SEM images.

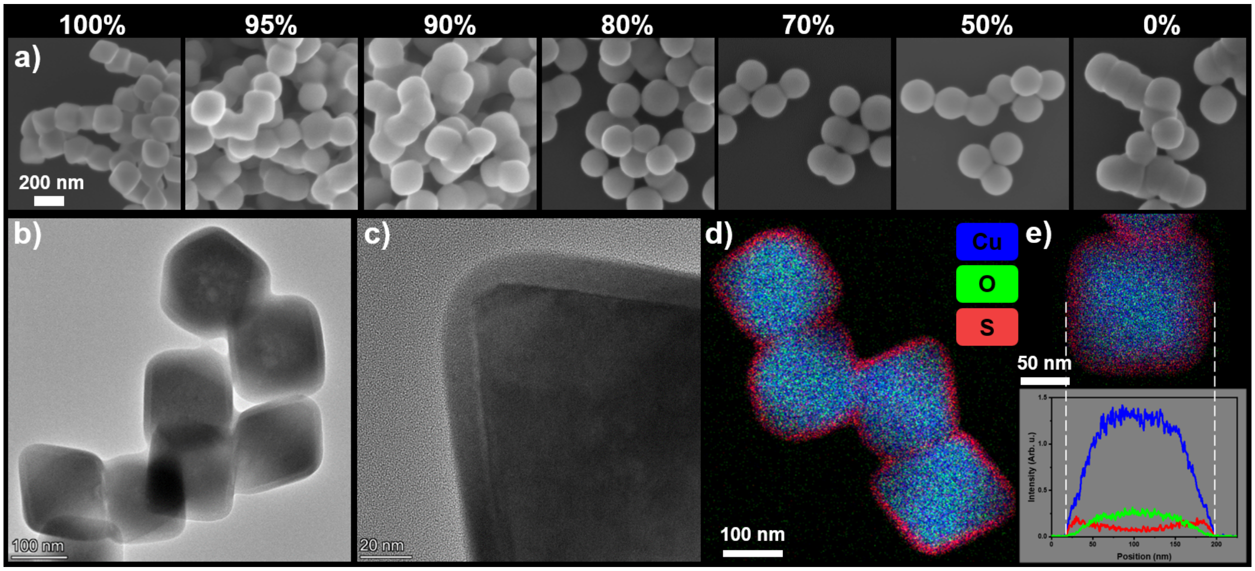

Figure S12. Effect of solvent composition on the morphology of the sulfidized particles. SEM images as a function of the ethanol v/v % in the ethanol:water mixture (a). Scale bar represents 200 nm for all SEM images. TEM images (b, c) and elemental maps Cu_2O/Cu_2S core/shell nanooctahedra synthesized in 100% ethanol (d, e). Elemental line profile across an individual core/shell octahedron (e).
